# Exploring the role of the inflammasomes on prostate cancer: Interplay with obesity

**DOI:** 10.1007/s11154-023-09838-w

**Published:** 2023-10-11

**Authors:** Jesús M. Pérez-Gómez, Antonio J. Montero-Hidalgo, Antonio C. Fuentes-Fayos, André Sarmento-Cabral, Rocio Guzmán-Ruiz, María M. Malagón, Aura D. Herrera-Martínez, Manuel D. Gahete, Raúl M. Luque

**Affiliations:** 1 Maimonides Biomedical Research Institute of Cordoba (IMIBIC), IMIBIC Building, Av. Menéndez Pidal s/n, 14004 Córdoba, Spain; 2 Department of Cell Biology, Physiology, and Immunology, University of Córdoba, Cordoba, Spain; 3 Hospital Universitario Reina Sofía (HURS), Cordoba, Spain; 4 Centro de Investigación Biomédica en Red de Fisiopatología de la Obesidad y Nutrición, (CIBERobn), Cordoba, Spain; 5 Endocrinology and Nutrition Service, HURS/IMIBIC, Córdoba, Spain

**Keywords:** Obesity, Prostate cancer, Inflammasome, Adipose tissue, IL-1β, NF-κB

## Abstract

Obesity is a weight-related disorder characterized by excessive adipose tissue growth and dysfunction which leads to the onset of a systemic chronic low-grade inflammatory state. Likewise, inflammation is considered a classic cancer hallmark affecting several steps of carcinogenesis and tumor progression. In this regard, novel molecular complexes termed inflammasomes have been identified which are able to react to a wide spectrum of insults, impacting several metabolic-related disorders, but their contribution to cancer biology remains unclear. In this context, prostate cancer (PCa) has a markedly inflammatory component, and patients frequently are elderly individuals who exhibit weight-related disorders, being obesity the most prevalent condition. Therefore, inflammation, and specifically, inflammasome complexes, could be crucial players in the interplay between PCa and metabolic disorders. In this review, we will: 1) discuss the potential role of each inflammasome component (sensor, molecular adaptor, and targets) in PCa pathophysiology, placing special emphasis on IL-1β/NF-kB pathway and ROS and hypoxia influence; 2) explore the association between inflammasomes and obesity, and how these molecular complexes could act as the cornerstone between the obesity and PCa; and, 3) compile current clinical trials regarding inflammasome targeting, providing some insights about their potential use in the clinical practice.

## Background

Prostate cancer (PCa) is a heterogeneous, complex, and strongly hormone-dependent disease that represents the second most frequently diagnosed cancer among the male population (14.1%), and the fifth leading cancer-related death in men worldwide among all cancer types (6.8%) [1]. In this context, the development and progression of PCa are controlled by different mechanisms that remain still not fully understood. Established risk factors for PCa development include age, ethnicity, and genetic background, while others like hormone levels, obesity, or prostatic inflammation have not been completely elucidated yet [2–4]. Inflammation is frequently associated with carcinogenesis and is also considered a common hallmark and a potential risk factor for the development of diverse malignancies through several mechanisms, including soluble molecules provided by the tumor microenvironment, extracellular matrix remodeling, angiogenesis, invasion, metastasis, and even epithelial to mesenchymal transition (EMT) [5, 6]. In fact, about 20% of all cancers are linked to some form of inflammation [7].

In the case of PCa, chronic inflammation has been proposed as a potential link between environmental stimuli and tumor occurrence. In this sense, the patients with high-grade inflammation surrounding malignant glands in radical prostatectomy specimens had significantly more biochemical PCa recurrence than patients with low-grade inflammation [8]. In fact, different studies have evaluated the relation between the onset of prostate gland abnormalities and some degree of inflammation, showing a high prevalence of mild chronic inflammation in benign prostate hyperplasia (BPH) and PCa samples [9–13].

In addition, genetic polymorphisms association studies highlighted that seven inflammation-related genes (*CXCL12*, *IL4*, *IL6*, *IL6ST*, *PTGS2*, *STAT3*, and *TNF*) may play a role in the development and aggressiveness of PCa [14]. However, during the last years, subsequent metanalyses have also revealed controversial results [15–18], suggesting that a positive association between prostate inflammation and PCa could be a consequence of detection bias [19], or that even such relation could be inverse [20]. In addition, reinforcing the idea that inflammation plays a key role in PCa progression, a type of proliferative inflammatory atrophy (PIA) has been associated with increased chronic inflammatory cell infiltration. These lesions are considered a possible precursor of high-grade prostatic intraepithelial neoplasia (PIN) and PCa by some authors and are usually found in the peripheral zone of the prostate, wherein PCa more frequently occurs [15, 21].

Therefore, increasing evidences suggest that inflammation could influence PCa development, and that immune cells could act as primary drivers. In fact, changes in the proportions and ratios of different immune cells populations are observed throughout different stages of PCa compared with healthy tissue [22]. Hence, it has been hypothesized that specific inflammatory infiltrate components are involved in PCa initiation or progression through the recruitment of additional immune cells and subsequent production of cytokines that ultimately creates a tumor microenvironment that promote cell replication, angiogenesis, EMT, migration, and metastasis [3, 23]. In this sense, initiation of prostatic inflammation could be driven by multiple and diverse agents, including uric acid (due to urine reflux), hormonal changes, physical damage, dietary components like heterocyclic amines, or microorganism presence [24].

In this context, a study focusing on the analysis of the urinary microbiome in urine samples suggested a prevalence of pro-inflammatory bacteria uropathogens in the urinary tract of men with PCa [25]. In fact, there is a close relationship between microorganism-originated inflammation, oxidative stress, and genomic instability. Indeed, bacterial prostatic colonization and/or dysbiosis could trigger signaling pathways that lead to immune cell infiltration, which can contribute to chemo-cytokines, growth factors, and ROS production [26]. In this scenario, it has been proposed that a prolonged oxidative stress environment could generate DNA damage (while decreasing DNA repair ability), which, added to the proinflammatory microenvironment, could derive into the above-mentioned PIA, followed by low or high-grade PIN, and ultimately, PCa [27]. Additionally, genetic changes and epigenetic background could drive chronic inflammation in the prostate epithelial cells [28].

Furthermore, weight-related disorders, including overweight or obesity have been postulated to mediate prostate inflammation [29]. Specifically, obesity is a metabolic pathogenic state that courses with systemic chronic low-grade inflammation and high circulating levels of inflammation-related markers (e.g., leptin, IL-6, TNF), which may affect tumor growth [30, 31]. In fact, adipocyte hypertrophy during obesity has been demonstrated to cause dysfunction and inflammation of the adipose tissue (AT) by increasing the secretion of pro-inflammatory cytokines [31, 32]. The relationship between obesity and PCa has been widely explored during the last decade [33–36]. Different studies reveal somewhat conflicting results, but there seems to be a general trend showing that obesity increases the risk of undergoing aggressive PCa [4]. Notwithstanding both pathological conditions often coexist, the direct molecular mechanisms that connect obesity and PCa are still poorly known.

In this context, inflammasomes have emerged as master regulators of inflammation by enabling the maturation of pro-inflammatory molecules through caspase-1 activation [37]. Evidences links the dysregulation of different types of inflammasomes (or some of their components) with several types of cancer, such as glioblastoma, hepatocellular carcinoma, mesothelioma, colorectal, gastric, lung, breast cancers, head and neck squamous cell carcinoma, oral squamous cell carcinoma or melanoma [38–40].

This review aims to summarize the current knowledge on the contribution of the different components of the inflammasome in PCa, incorporating, for the first time, its relationship with obesity and specifically with AT depots, as well as the potential of these inflammatory complexes as therapeutic tools to tackle PCa.

## The inflammasomes

As the first line of defense, the innate immune system has evolved to detect and react to a wide variety of insults derived from external and self-signals. Those stimuli include conserved structures among microorganisms, which are called pathogen-associated molecular patterns (PAMPs), and endogenous molecules associated with components of host cells that are associated to cell damage, stress, or death, termed damage-associated molecular patterns (DAMPs, also known as alarmins) [41]. These signals include reactive oxygen species (ROS), oxidized and/or methylated DNA, the presence of abnormal proteins in certain cellular compartments, increased levels of ATP, free fatty acids, ceramides, homocysteine, and calcium or cholesterol crystals, among others [42].

To recognize these signals, the innate immune system possesses, among its immunological arsenal, the germline-encoded pattern recognition receptors (PRRs), which can be classified into membrane-bound and cytosolic sensors. Membrane-bound sensors include Toll-like receptors (TLRs) and C-type lectin receptors (CLRs), while cytosolic sensors comprise the nucleotide-binding oligomerization domain-like receptors, or NOD-like receptors (NLRs), and the retinoic acid-inducible gene 1 (RIG-1)-like receptors (RLRs) [43–45]. Particularly, the NLR family comprises 22 genes that are characterized by the presence of a central NACHT domain, along with other variables such as PAAD/DAPIN (PYD) and caspase activation and recruitment (CARD) domains, both involved in homotypic protein-protein interactions [46].

**Fig. 1:**
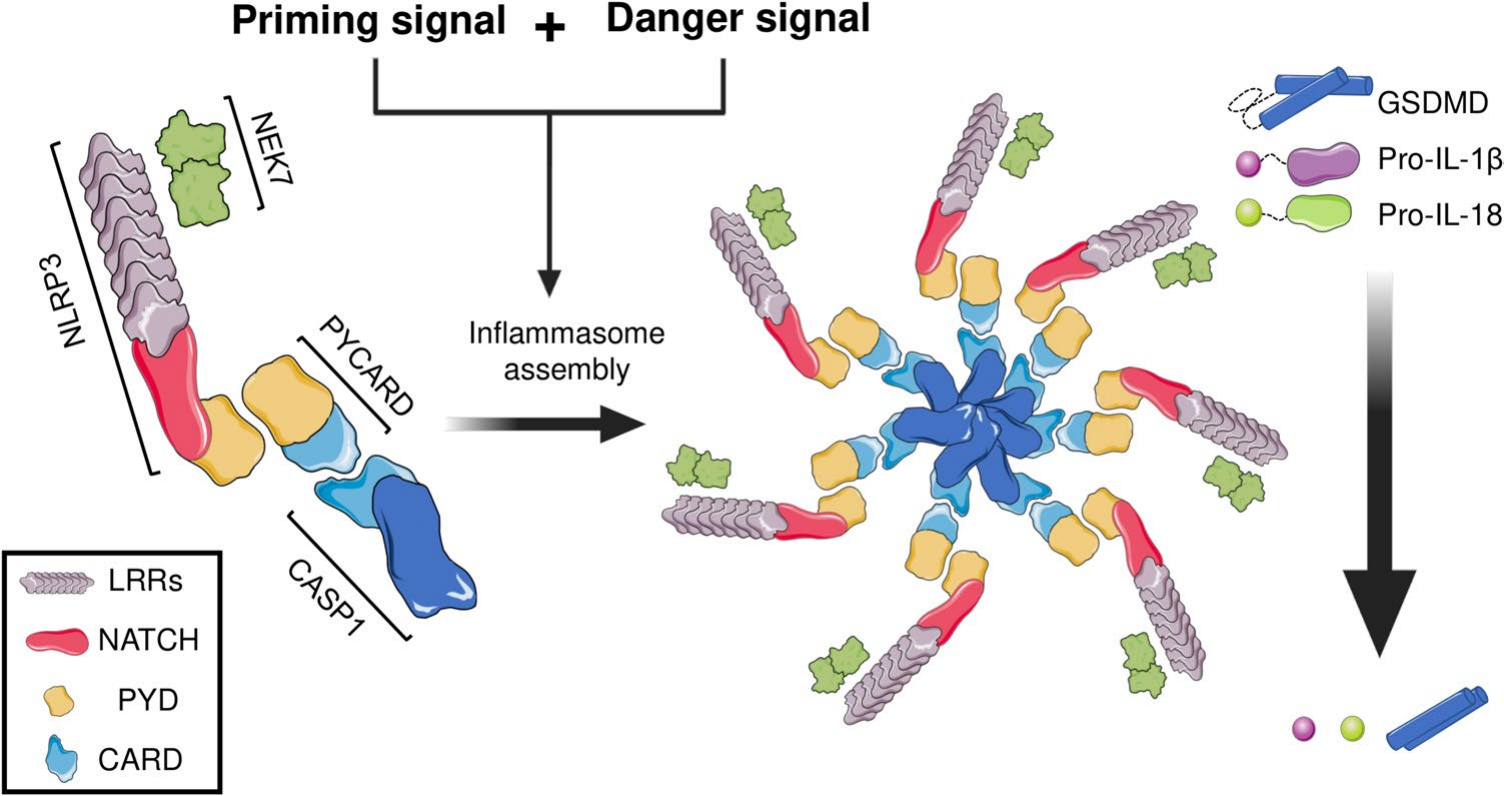
*General NLRP3 inflammasome structure and activation.* After priming and danger signal combination, inflammasome monomers composed by sensor molecular (NLRP3), assembly and activity regulator (NEK7), molecular adaptor (PYCARD) and enzymatic subunit (zymogenic CASP1) oligomerize forming a heptameric macromolecular platform that enables proteolytic autoactivation of CASP1 and processing of three proinflammatory molecules: pyroptotic-related membrane pore subunit Gasdermin D (GSDMD), and interleukins-1-β and-18

Inflammasomes are cytosolic complexes capable of triggering an inflammatory response through the autoactivation of caspase-1, which leads to proteolytic cleavage, maturation, and secretion of pro-inflammatory molecules, including the interleukin-1β (IL-1β) and interleukin-18 (IL-18). These two cytokines are synthesized as the inactive precursors pro-IL-1β (p31) and pro-IL-18 (p24), which are finally processed through inflammasome activation to their mature form IL-1β (p17) and IL-18 (p18), respectively [47, 48]. While pro-IL-18 is constitutively expressed, pro-IL-1β expression is often upregulated after ligand-mediated activation of TLRs or NLRs family members, constituting a previous and limiting step for the activation of certain inflammasomes activation, termed priming [49]. In addition, a third product derived from inflammasome activation, gasdermin D (GSDMD), has been recently described (Fig. 1). Once cleaved, this protein is able to initiate an inflammation-mediated cell death process known as pyroptosis [50].

Inflammasomes usually include 3 types of proteins in a heptameric conformation: 1) a member of the NLR family protein that acts as the stimuli sensor; 2) the molecular adaptor ASC (also known as PYCARD, containing both PYD and CARD domains), which serves as a scaffold; and 3) the inactive zymogen form of the caspase-1, recruited for final autoactivation. According to the specific inflammasome composition, the molecular adaptor ASC may not be necessary since the sensor protein could directly interact with caspase-1 [51]. There are different types of inflammasomes, named according to their sensor component. Of these, NLRP1, NLRP3, NLRC4, AIM2, and pyrin (a PYD domain-containing protein) inflammasomes are considered canonical, while recent studies have identified non-canonical or atypical inflammasomes existence, like NLRP2, NLRP6, NLRP7, NLRP12, and IFI16 inflammasomes [52–57]. In addition, a fourth component, NEK7, has been recently discovered to be essential for oligomerization and triggering of some types of inflammasomes [58]. Remarkably, CASP8 activating inflammasomes have been described, and active forms of IL-33 or IL-37 have also been proposed to be produced by certain inflammasomes types [59, 60].

Distinct inflammasomes are activated in response to a plethora of stimuli with different action mechanisms. Among them, the NLRP3 inflammasome is currently the most fully characterized [61, 62]. The proposed NLRP3 inflammasome activation mechanism follows a two-step model (Fig. 1). First, a signal acts as the priming step and licenses inflammasome by upregulation of NLRP3, IL-1β, and IL-18 through downstream activation of TLRs and MyD88, and subsequent NF-κB pathway activation, in an NLRP3/NEK7-dependent mechanism. Second, a hazardous signal is sensed by NLRP3, allowing inflammasome assembly and caspase-1 activation (Fig. 1). The activation of NLRP3 inflammasome across a wide diversity of stimuli allow the convergence of these signals into a common signaling mechanism [63]. The exact convergent process is unclear, but proposed mechanisms include mitochondrial ROS production, ATP-dependent potassium efflux, mitochondrial DNA release, lysosomal cathepsins release, changes in cell volume, and mitochondrial cardiolipin translocation [64–66].

Importantly, dysregulation of inflammasome complexes has risen as a critical molecular element in metabolic and cancer pathologies in humans [67–70]. Specifically, in the context of cancer development and progression, inflammasomes could play opposite roles like tumor-suppressor functions (by inducing cell apoptosis), or tumor-promoting events (by favoring secretion of pro-inflammatory cytokines). Such heterogeneous effects within the tumor microenvironment could be mediated by the tumor cells, the tumor-infiltrating myeloid cells, or both. Furthermore, despite the inflammasomes has been typically associated with myeloid cells such as monocytes, its activation has also been demonstrated in non-myeloid cells [37, 71]. Of special interest, functional inflammasomes have been found in prostate epithelial cells as well as in adipocytes, suggesting that they could be an essential supramolecular mediator in the potential crosstalk between both cell types under normal and tumoral conditions [71, 72].

## Inflammasomes in prostate cancer

A growing body of evidence suggests inflammation as a potential element in PCa initiation and progression. Thus, the identification of inflammation regulators and associated molecules may provide an opportunity to understand the role of inflammation in PCa. In this context, the inflammasomes has emerged as a crucial player in the inflammatory state. In the next sections, we will discuss the current knowledge of inflammasome components and their potential role as regulators in PCa (listed in Table 1) pathophysiology,as well as their potential association with obesity.

**Table 1:** Inflammasomes components dysregulations evidences in prostate cancer

**Component**	**Annotation**	**Model**	**Ref**
**TLR3**	NF-κB activation Apoptosis and angiogenesis regulation Overexpression and PCa recurrence correlation	LNCaP, PC-3, DU145 LNCaP, PC-3 Human PCa cohort	[67] [78] [69]
**TLR4**	SNPs positive association with PCa risk Tumoral mRNA and protein decreased levels Decreased levels/ IL-1β overexpression through NF-κB and MyD88 pathway Vasculature induction Chemoresistance NF-κB activation-dependent migration	Human PCa cohorts DU145, PC-3 Human PCa cohort/DU145 Human PCa cohort PC-3 PC-3, DU145	[70–72] [75] [76] [77] [78] [79]
**TLR9**	Overexpression, poor progression-free survival association Increased invasion and metastasis capacity	DU145, PC-3, LNCaP, C4-2B, Human PCa cohort PC-3	[80, 81] [82]
**AIM2**	Decreased mRNA levels AIM2 inflammasome priming and activation under hypoxic conditions via NF-κB	DU145, PC-3, LNCaP, Human PCa cohort/ BPH-1, PC-3	[64] [88]
**NLRP3**	NLRP3 inflammasome activation under hypoxic conditions	BPH-1, PC-3	[88]
**PYCARD**	Promoter methylation	LNCaP, LAPC-4, MDAPCa2b, DU145, PC-3, Human PCa Cohort	[108, 109]
**CASP1**	Decreased levels of active form	Human PCa Cohort	[116]
**IL-1β**	Polymorphisms associated with increased cancer risk Polymorphisms associated with decreased cancer risk Progressive immunohistochemical loss Growth inhibition after external administration Increase HIF-1α through NF-κB activation	Human PCa Cohort Human PCa Cohort Human PCa Cohort LNCaP, PC-3, DU145 Human PCa Cohort	[122] [123] [124] [125] [134]
**IL-18**	Overexpression	PC-3, DU145, Human PCa Cohort	[141, 142]

### Toll-like receptors (TLRs)

TLRs are membrane-bound danger-sensing receptors tightly associated with innate immune response. In humans, ten TLRs with differential specificity for distinct types of insults have been identified [43]. Despite TLRs constitute a group of well-characterized pro-inflammatory signaling pathways, recent advances have linked these receptors to inflammasome activation. In fact, previous studies have shown that TLR signaling is not only an enhancer of inflammasome response but also indispensable for certain inflammasomes types [51]. The most explored TLRs in the PCa context are TLR3, TLR4, and TLR9.

#### TLR3

TLR3 is an intracellular receptor located in endosomal membranes that is involved in the recognition of nucleic acids. TLR3 has been detected at mRNA and protein levels in different PCa cell models (LNCaP, PC-3, and DU145 cell lines). Moreover, TLR3 stimulation has been shown to increase the expression of inflammatory molecules through activation of the NF-κB pathway leading to the recruitment of specific leukocyte populations [73]. In addition, TLR3 has been proved to regulate apoptosis and angiogenesis in LNCaP and PC-3 cells through HIF-1α, a protein highly related to inflammasome activation as we will discuss below [74]. Furthermore, the expression of TLR3 has been corroborated in human samples, finding a significant and positive correlation between TLR3 levels and PCa recurrence [75].

#### TLR4

TLR4 recognizes lipopolysaccharides and constitutes the most studied TLR in PCa. Several genetic polymorphism studies have been performed correlating the presence of single nucleotide polymorphisms (SNPs) in the TLR4 gene with PCa risk development, showing conflicting results. Some authors concluded that inherited polymorphisms of TLR4 are associated with increased risk of PCa [76–78], while others have established that those polymorphisms were not significantly associated [79, 80]. Such differences could be explain due to the different number of patients included in and/or ethnic differences of the population studied. TLR4 has also been detected (at mRNA and protein levels) in distinct PCa cell lines (DU145 and PC-3) and tumor samples, despite at significantly lower levels as compared with BPH tissue [81]. Gatti and co-workers demonstrated that TLR4 levels decreased as the histopathological grade of tumors increased, and that such reduction was directly associated with epithelial, but not stromal, prostate cells. Interestingly, the same authors demonstrated that TLR4 activation in DU145 cells courses through NF-κB and MyD88-dependent pathways, resulting in increased expression of IL-1β mRNA (priming effect) and increased protein levels after TLR4 induction [82]. Additionally, TLR4 activation seems to be mediated by peroxiredoxin 1 (an antioxidant enzyme elevated and secreted by tumor prostate cells) conferring chemoresistance in PC-3 cells [83, 84]. Additionally, a recent work by Zhonghua and collaborators demonstrated that S100A9, classically considered an alarmin, promotes cancer cell invasion in PC-3 and DU145 cells through TLR4 binding in an NF-kB-dependent mechanism [85].

#### TLR9

On the contrary, TLR9, is a receptor associated with inner cell membranes. TLR9, which recognizes CpG oligonucleotides, has been detected at the protein level in DU145, PC-3, LNCaP, and C4-2B, as well as in human samples of PCa [86]. Indeed, TLR9 levels were associated with poor progression-free survival in PCa patients [87]. In addition, TLR9 up-regulates cyclooxygenase 2 (COX-2) expression in PC-3 cells through NF-κB activation, which has been suggested by some authors to be implicated in tumor invasion and metastasis [88].

Taken together, TLRs activation itself, along with distinct inflammasome licensing and pathway cross-talking, might induce early immune responses in the tumor microenvironment that could influence the fate of PCa progression.

### Sensor component

The essential difference among inflammasome subtypes is the sensor protein, which gives specificity against stimuli that trigger inflammasome assembly. In the next subsections, we compile current information about AIM2 and NLRP3 components, the most explored in the context of carcinogenesis and PCa.

#### AIM2

*AIM2* encodes a cytosolic member of the IFI20X/IFI16 (PYHIN) protein family. This protein associates with cytoplasmic double-stranded DNA (dsDNA) via a HIN200 domain, while its PYD domain interacts with the adapter molecule ASC to activate caspase-1, conforming the AIM2 inflammasome [89–91]. AIM2 inflammasome has been postulated to be activated in response to exogen and self-derived dsDNA, enabling pro-inflammatory cytokines production [71]. Moreover, *AIM2* has been associated with different tumors subtypes wherein classically has been described as a tumor suppressor. For instance, Aim2^−/−^ models showed a higher susceptibility to develop colon cancer compared to control mice, although cytokine production and inflammasome activation is not compromised. In this sense, it has been postulated that *AIM2* could be exerting this tumor suppressor role through the regulation of PTEN on the ATK pathway, and by the activation of the proto-oncogene c-Myc, in a very specific context where gut microbiota and the AIM2 dsDNA sensing capacity could be crucial, although mechanistic details are lacking [92]. However, it should be mentioned that a prooncogenic role for *AIM2* has also been described in squamous cell carcinoma, and endometrial cancers [93].

*AIM2* is expressed in human normal prostate epithelial cells and its expression, along with that of other key inflammasome components (i.e., pro-caspase-1 and pro-IL-1β), is potentiated when prostate cells are primed with INFα, β, and γ [71]. Furthermore, steady-state levels of AIM2 are significantly lower in young epithelial prostate cells than in senescent cells. Accordingly, AIM2 levels were also significantly lower in normal prostate epithelial cells compared with the BPH-1 cell line, suggesting that the accumulation of prostate cells with a senescent secretory phenotype (also refer as SASP) may play an important role in BPH initiation [71]. Surprisingly, *AIM2* mRNA levels were significantly lower in various PCa cell lines (DU145, PC-3, and LNCaP) as well as in prostate adenocarcinoma samples compared with normal prostate epithelial cells [71, 94]. Notably, it has been proven that primed AIM2 inflammasome is activated in the presence of cytosolic dsDNA in normal epithelial prostate cells and PC-3 cells [71].

Panchanathan and cols*.* recently demonstrated that hypoxic conditions often associate with inflammation in solid tumors, and, specifically with tumor promotion, resistance to therapy, and malignant progression in PCa [95]. Thus, hypoxic conditions prime and activate AIM2 inflammasome in human normal epithelial cells, besides promoting the activity of those previously primed by cytosolic dsDNA [95]. In line with this, hypoxia also increases caspase-1 activity levels in normal prostate epithelial cells, BPH-1 and in PC-3 cell line via NF-κB pathway activation. Similar results were obtained in the myeloid cell line TPH-1 [95]. Furthermore, hypoxia and inflammation keep a feed-forward loop relation in prostatic tumors, enabling tumor promotion, malignant progression, resistance to cancer therapy, and consequently poor outcome for PCa patients [96, 97]. With all this evidences, we could conclude that AIM2 may represent a protein with an important role in oncogenesis in a tissue and context-dependent manner. Therefore, a special effort should be made to clarify its potential consequences in PCa development.

#### NLRP3

NLRP3 inflammasome is characterized by the ability to respond to a wide spectrum of stimuli. However, this inflammasome subtype rarely responds to a single signal. NLRP3 inflammasome possesses a double-step initiation model that requires previous priming, resulting in *NLRP3* and *IL1B* genes upregulation [63]. This dual signal requirement may act as a checkpoint mechanism to avoid accidental or uncontrolled NLRP3 activation. In cancer research, NLRP3 inflammasome has been also widely explored, appearing to be relevant in such diverse tumors as glioblastoma, and colorectal or liver cancers. Moossavi and colleagues recently summarized the known role of NLRP3 inflammasome in cancer [39]. However, scarce, and conflicting information is available in PCa. Some studies reveal that this sensor component can be detected in several prostate-derived cell lines (RWPE-1, PZ-HPV-7, LNCaP, Ln3, 22Rv1, PC-3, and DU145) at mRNA and protein levels, while other studies indicate that NLRP3 protein and transcripts were undetectable or extremely low in PC-3 cells [98]. A recent study showed that NLRP3 expression level was significantly high (at mRNA and protein levels) in various PCa cell lines (i.e., DU145, 22Rv1, PC-3, LNCaP) compared with the healthy cell line WPMY-1. In humans, PCa samples have been shown to exhibit heterogeneous immunostaining for NLRP3 compared with normal adjacent tissue [99], while other studies indicate that NLRP3 expression levels are significantly high in PCa tissues, wherein this overexpression is correlated with clinical parameters such as TNM stage and lymph node invasion [98].

Panchanathan and cols. also demonstrated a post-transcriptional stabilization of NLRP3 by hypoxia. Apart from acting as a priming agent in human normal prostate cells, benign BPH-1, and tumor PC-3 cell lines, hypoxia also resulted in enhanced activation of NLRP3 inflammasome [95]. Unlike that observed in the case of AIM2 inflammasome, hypoxia was not sufficient to prime or activate NLRP3 inflammasome in the myeloid cell line TPH-1 [95]. Finally, recent works have demonstrated that blocking NLRP3 pathway in PC-3 cells and PCa-derived xenografts have a significant impact on tumor progression by disrupting cell proliferation, migration, and/or inducing apoptosis [98, 100, 101].

Considering these results, it might be suggested that among the complex tumor microenvironment, distinct stimuli could trigger specific inflammasomes in a cell type-specific manner, which could finally affect the development of prostate inflammatory-related pathologies, including cancer.

### Molecular adaptor: PYCARD (ASC)

Apoptosis-associated Speck-like protein containing a CARD (ASC), also referred as PYCARD, is a molecular adaptor necessary for assembling different types of inflammasomes. Through PYD and CARD domains, PYCARD participates in canonical (NLRP3, AIM2) and non-canonial (NLRP6, NLRP7, NLRP12, IFI16) inflammasomes [51]. Even though some sensor components like NLRP1 harbor domains that enable direct interaction with caspase-1, the presence of the PYCARD adaptor seems to be crucial to reach the maximum capacity of inflammasome [102].

PYCARD has been one of the most studied inflammasome components as it was first described as a methylation-associated silenced gene in human breast cancer cells [103]. Furthermore, PYCARD silencing and methylation status has been reported in several other neoplastic diseases such as colorectal, lung and ovarian cancer, melanoma, neuroblastoma, and glioblastoma [104–109], and has typically been supposed to act as a tumor suppressor implicated in caspase-mediated apoptosis (involving predominantly CASP8 and CASP9), and mitochondrial translocation of BAX coupled to activation of CASP9, CASP2, and CASP3 [110, 111]. However, since inflammasomes were discovered, ASC has been proposed to be implicated in tumor development/progression through different indirect mechanisms. Thus, in gastric cancer, it has been suggested the existence of potential signaling crosstalk between the activation of ASC inflammasomes (through NF-κB) and CASP8 apoptotic machinery in an intricate mechanism [112]. This likely dual role of PYCARD [apoptotic/tumor-suppressor vs. pro-inflammatory/oncogene (as happened with the above-mentioned AIM2)] in different cancer types has been recently reviewed [113].

In PCa, methylation of PYCARD has also been demonstrated in several tumor cell lines, including LNCaP, LAPC-4, MDAPCa2b, DU145, and PC-3 [114, 115], but not in the benign prostate epithelial cell line BPH-1, or normal prostate epithelial cells. Furthermore, Collard and cols*.* showed similar methylation trends of PYCARD with 64% of methylation in high-grade PIN samples examined, 65% in cancer specimens, and 28% in the adjacent normal tissue, while all normal donor prostate samples presented unmethylated *PYCARD* promoter [114]. However, further investigation is needed to unveil the intricate role of this multifunctional protein within this tumor pathology.

### Caspase 1

The *CASP1* gene encodes for caspase-1, a member of the cysteine-aspartic acid protease family that plays a central role in the execution-phase of cell apoptosis and the proteolytical activation of the inactive precursor of pro-inflammatory molecules as it is the enzymatic component of inflammasomes. Caspase-1 has recently gained attention as it may play a crucial role in human carcinogenesis [116]. In fact, several studies demonstrated a significant downregulation of *CASP1* expression in different types of cancers, including renal, breast, colon, or ovarian cancer, while upregulation has been reported in pancreatic tumors [117–121].

In the case of PCa, immunohistochemical analysis showed significantly decreased levels of the active form of CASP1 protein compared with the normal gland [122]. A constitutive expression level of the proenzyme form of CASP1 in human PCa cell lines (PC-3, DU145, and LNCaP) has been also reported [122]. The loss of *CASP1* has also been proposed as a required step in the dysregulation of apoptotic control during PCa development [122, 123]. Recently, Chang and cols*.* demonstrated that reduced *in vivo* levels of CASP1 may be due to a negative modulation effect of cytochrome P450-1B1 (CYP1B1) [124]. In line with this, the authors also found an inverse correlation between CYP1B1 and caspase-1 in human tissue specimens [124].

### Inflammasome targets

#### IL-1β

IL-1β is a major upstream cytokine that controls the local pro-inflammatory status and can be produced by monocytes, fibroblast, and epithelial cells as a paracrine or autocrine signal. IL-1β is one of the seven ligands included in the IL-1 family, which require inflammasome action to reach the mature and active form that binds to its heterodimeric IL-1RI/IL-1RAcP receptor on target cells to initiate IL-1β signaling [125]. IL-1β recognition trough IL1R leads to different associated kinases family (IRAK) assembly around Myd88, forming other types of macromolecular complexes (myddosomes) that may play an important role in myeloid malignancies and in the adaptative resistance to various forms of cancer therapy [126].

Due to IL-1β key role in the inflammatory response, its production affects the balance established between protective immunity and destructive inflammation. However, the role of IL-1β in tumor biology remains controversial. Some authors have proposed that IL-1β is able to induce tumor angiogenesis and metastatic spread, while an antitumor role has also been suggested [127–129]. In this sense, the role of IL-1β in PCa is still unclear. Different IL-1β polymorphisms have been associated with either increased or decreased PCa risk, providing additional evidence that this cytokine may play an important role in PCa etiology [130, 131]. Notably, Ricote and cols*.* demonstrated the immunohistochemical presence of IL-1β in 75% of normal prostate samples, in 42.86% of BPH samples, and in 31.25% of PCa samples with low Gleason grade, while no positive immunoreactions were observed in PCa samples with high Gleason grade, suggesting that a progressive loss of IL-1β is associated with more aggressive pathologies [132]. In line with these results, external reestablishment of IL-1β provokes growth inhibition in LNCaP and androgen-independent PC-3 and DU145 cell lines [133]. Furthermore, in LNCaP cells, IL-1β exposure is also able to decrease PSA production, Androgen Receptor (AR) mRNA/protein levels, and causes antiandrogen bicalutamide resistance after a few passages, linking the cytokine with castration resistant prostate cancer (CRPC) development [134]. Chang and cols*.* identified IL-1β as the HS-5 bone marrow stromal cell-secreted paracrine factor responsible for AR mRNA downregulation in C4-2 PCa cell line (LNCaP derived subcutaneous xenograft tumor of castrated mouse) through upregulation of p62, a multifunctional adaptor needed for NF-κB pathway activation [135].

A body of evidence points out that IL-1β exerts its effects on PCa through the NF-κB pathway [133, 135]. NF-κB is generally associated with antiapoptotic and pro-inflammatory effects, and recent evidence suggests that NF-κB may have anti-inflammatory roles and promote apoptosis during tumor progression [136, 137]. NF-κB is constitutively activated in diverse PCa cell lines and human prostate adenocarcinoma, while trans-localization into the nucleus of PCa cells positively correlates with a high Gleason score, biochemical recurrence, and metastasis due to its transcriptional regulation activity [138, 139]. A detailed list of proposed targeted genes regulated by NF-κB in PCa is available [138]. Of note, interleukin-6 (IL-6), a cytokine widely studied in PCa, is upregulated by NF-κB action and it has been proposed as a key factor in the transition from hormone-dependent to CRPC [140]. In fact, NF-κB pathway activation itself seems to be sufficient to allow prostate and PCa to grow in an androgen-independent manner through AR activity regulation, confer protection against apoptosis, and participate in the induction of metastatic phenotype, thus being considered the most important effector of pro-inflammatory processes involved in PCa pathogenesis by various authors [138, 141, 142].

Notably, NF-κB activation also produces upregulation of IL-1β, therefore causing a positive feedback loop [138]. Hypoxia regulator HIF-1α induced by Prx1 also promoted NF-κB activity, which has the potential to perpetuate Prx1 induction of angiogenesis in PCa. In addition, this mechanism occurs through TLR4-dependent activation, which has been related to the inflammasome priming phenomenon [83]. In this regard, it has been suggested that hypoxia can induce AIM2 and NLRP3 inflammasome activation and consequent IL-1β production through stimulation of the NF-κB pathway in human normal prostate, PC-3, and myeloid THP-1 cell line [95]. Notably, the expression of the master regulator of hypoxia HIF-1α increases in response to IL-1β through NF-κB activation, and IL-1β itself can stabilize HIF-1α even under normoxia conditions, through a ROS-dependent mechanism [143, 144]. Moreover, hypoxia strongly induced the expression of PD-L1 in 22Rv1, DU145 and PC3 cell lines, while HIF1-α knockdown and chrysin treatment (an inhibitor of HIF1-α stability) decreases PD-L1 protein levels (except for 22Rv1) [145]. Interestingly, it seems to be a mutual crosstalk, between the exploited PD-1/PD-L immunosuppressive system, that drives immune scape and tumor growth, and inflammasomes. For example, it is known that AIM2 can regulate PD-L1, and that IL-1β is able to increase PD-L1 at the membrane of cancer cells and CAFs. A review has recently been published addressing this connection [60].

In a recent work, Tran and colleagues explored the link between HIF1-α and oncogenic AR signaling in CRPC development through transcriptomic profiles, concluding that both pathways occur in an independent manner [146]. However, it is feasible that hypoxia and therefore increased HIF1-α levels could alter AR signaling and promote CRPC development through a complex network of inflammasome-derived cytokines such as IL-1β. Interestingly, ROS, which are also capable to stabilize HIF-1α, have been proposed to be necessary, but not sufficient, for triggering inflammasome activation [51, 144]. Indeed, a double role for ROS has been suggested concerning inflammasomes functioning: first, ROS are thought to be able to direct priming and triggering some types of inflammasomes; additionally, ROS can also exert an indirect inductor effect through cytoplasmic proteins that control inflammasomes activity. Moreover, ROS has been proposed as a likely common signal throughout different stimuli that can converge in inflammasome activation [147].

In a hypoxic-ROS enriched microenvironment, DNA damage and repair mechanisms disfunction, could contribute significantly to the establishment of tumor origin. For example, Rar50 (a major constituent of the MRN-DSBs [Mre11-Rad50-Nbs1 Double Strand Breaks] complex), is needed for the NF-kB activation and pro-IL1-β production. Moreover, Rad50 is also implied in viral and bacterial dsDNA recognition, elements that trigger certain inflammasomes types [148].

**Fig. 2:**
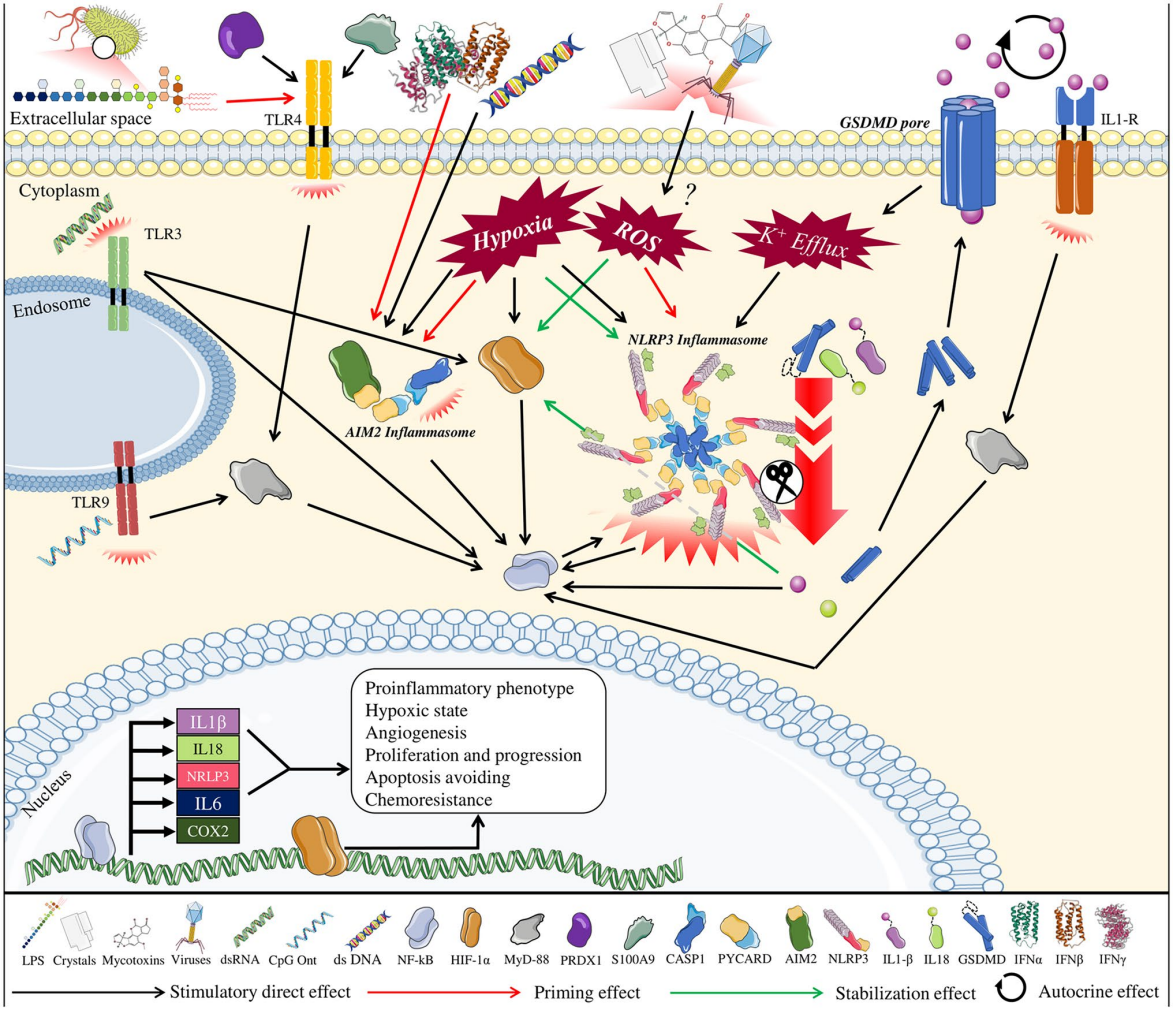
*The central role of inflammasomes and NF-kB signaling in prostate cancer.* Different types of inflammasomes are capable of activating and responding to a wide variety of stimuli such as pathogens (PAMPs) or stress situations intrinsic to the cell itself (DAMPs). The activation of the inflammasome is enhanced and shares some signaling in common with the hypoxia signaling pathway, which in turn exacerbates inflammasome activation, establishing multiple positive feedback loops. As a result, it exists a convergence in terms of signaling through NF-kB, which acts as a transcription factor of essential genes in the development and progression of prostate cancer toward more aggressive phenotypes

In addition, IFN signaling (released upon viral infection), which is able to prime and activate AIM2 inflammasome, interacts with the NF-κB pathway providing an additional link between inflammasomes and NF-κB and PCa [71, 137]. Of note, NF-κB signaling is a major inducer of SASP, which is related to inflammasome component AIM2 accumulation and BPH initiation [149]. Taken together, current knowledge places inflammasomes in a central position within a complex crossover of pathways orbiting around danger or stress signals, IL-1β and NF-κB (Fig. 2).

#### IL-18

IL-18 is a pleiotropic cytokine that plays a central role in inflammation and immune response by exerting several functions, mostly related to immunomodulation in macrophages, T-helper type I (Th1), and natural killer (NK) cells [150]. Like IL-1β, IL-18 reaches its active form after caspase-1 processing activity. IL-18 has been widely associated with antitumor effects in different types of cancer by immunosurveillance modulation through different immune cell types [151]. Despite its antitumor effects, IL-18 is expressed in PC-3 and DU145 cell lines after IFN-γ induction [152]. In line with this, PCa samples also exhibit IL-18 overexpression, being its levels associated with a better clinical outcome [152, 153]. However, IL-18 binding protein (IL18BP), a glycoprotein with a high affinity to IL-18 able to neutralize it, is also overexpressed in PCa human samples and some PCa cell lines under IFN-γ stimulation. Noteworthy, IL-18BP serum levels correlate with the Gleason score [151]. Taking all these evidences together, it could be suggested that these processes might be involved in a regulation mechanism of inflammasome assembly and caspase-1 activation affecting prostate and contributing to PCa development, which needs to be further investigated.

#### Gasdermin D

While most cytokines include an N-terminal sequence related to its secretion, members of the IL-1 family, including IL-1β and IL-18, do not possess any known sequence associated with extracellular release [154]. Recently, a novel cytokine releasing mechanism involving a third inflammasome product, Gasdermin D (GSDMD) has been discovered. GSDMD belongs to the gasdermin family, together with GSDMA, GSDMB, and GSDMC. This protein remains inactive until cleavage activation by several caspases (including inflammasome component caspase-1); once cleaved, GSDMD oligomerize on membranes through specific phospholipid interactions, forming pores. It has been suggested that low levels of GSDMD form functional pores, which is associated with long-term IL-1β release by still-living cells [155]. However, it has been proposed that at high GSDMD levels, cell membrane repair activity would not be sufficient to avoid cellular death, provoking the releasing of intracellular content causing a highly inflammatory uncontrolled death known as pyroptosis. Ultimately, GSDMD pores have been proposed to enable potassium efflux alteration, leading to NLPR3 inflammasome activation and to the potentiation of a positive feedback loop [156]. Such inflammatory cell death may play a dual role in promoting or inhibiting tumor cell growth in different cancer types, showing controversial results [157]. Particularly, GSDMD contribution to cancer development is poorly explored and has only been reported by Wang and cols*.* in gastric cancer, wherein GSDMD is downregulated among different gastric cancer cell lines by inhibiting the cyclinA2/CDK2 complex and arresting into the S/G2 phase transition [158]. This study also proposes GSDMD as a tumor suppressor acting through ERK, STAT3, and PI3k pathway inactivation. Although to date, GSDMD role in PCa is completely unexplored, Zhang and cols*.* recently demonstrated GSDME ability to induce pyroptosis in PC-3 cells through PKCδ/JNK signal activation [123].

## Obesity, adipose tissue, and inflammasomes in prostate cancer

According to World Health Organization (WHO), obesity is defined as a condition of excessive fat accumulation in adipose tissue (AT) due to a positive energy balance as a consequence of an increased energy intake and/or decreased energy expenditure [159]. Obesity has risen in the last decades as a major global issue, reaching pandemic proportions and becoming a real challenge for healthcare systems [160, 161]. Obesity is characterized by AT disfunction; this tissue constitutes a complex, heterogeneous, and metabolically active organ that has been classically proposed to comprise mature adipocytes and the stromal-vascular fraction (SVF), composed of preadipocytes, fibroblasts, vascular endothelial cells, and a variety of immune cells including macrophages, lymphocytes, mast cells, neutrophils, eosinophils, and foam cells [162–164]. Pathophysiologically, obesity triggers an inflammatory response orchestrated by adipocytes, then, milieu composition modification occurs (including extracellular remodeling and immune cell infiltration and activation) and, finally, a characteristic maintained/chronic low-inflammatory systemic state is established, a condition termed “metaflammation” by some authors [165]. Once established, this condition associates with comorbidities including type 2 diabetes (T2D), cardiovascular disease, atherosclerosis, hepatic steatosis, central nervous system dysfunction, dementia, and certain cancers, including PCa. In this context, AT is now considered an important mediator of systemic and local inflammatory responses, thus emerging as a key immunometabolic organ connecting lipid metabolism dysfunction and altered adipokine release, and distinct types of pathologies.

In that respect, inflammasomes exert a great impact on obesity and weight-related comorbidities. Specifically, Vandanmagsar and collaborators described increased mRNA expression of *NLRP3* and *IL-1β* (but not *ASC*) in AT from patients with obesity and T2D, and these levels correlated with glycemic state [166]. In addition, the presence of NLRP3 inflammasome components in a mouse model of diet-induced obesity was higher in AT macrophages (ATMs) and SVF than in mature adipocytes of epididymal fat tissue, which suggested a theoretical model based on positive feed-forward loops among adipocytes, macrophages, and T cells [166]. Increased inflammation levels due to increased caspase-1 activity and IL-1β production by ATMs have been also described in visceral adipose tissue from metabolically unhealthy patients [167]. 

NLRP3 inflammasome is activated by ceramides sensing, inducing IL-1β production through caspase-1 activation, contributing to obesity-induced inflammation and insulin resistance. In line with this, cholesterol crystals and palmitate (the most increased serum fatty acid in obesity) have also been categorized as sterile inflammation instigators through NLRP3 inflammasome activation [166, 168]. In contrast, oleate, linoleate, and ω-3 fatty acids (docosahexaenoic and eicosapentaenoic acids) showed inhibitory effects [169]. Moreover, increased glucose levels also contribute to a low-chronic inflammatory state and insulin resistance partly through NLRP3 inflammasome activation and IL-1β production in subcutaneous adipose tissue (SAT) [170]. NLRP3 inflammasome has also been reported to be activated under obesity conditions through a hypoxia-dependent mechanism [171]. NLRP3, but not NLRP1 inflammasomes, are regulated by hypoxia conditions in visceral adipocytes [172]. Moreover, a recent report identified HIF-1α activation in ATMs responsible for local and systemic IL-1β maintained production under obesity conditions through ceramides sensing [173]. Moreover, it has been demonstrated that High Fat diet induces insulin resistance through NLRP3 inflammasome activation, and by a subsequent IL-1β secretion and ROS production in mouse AT [174]. Furthermore, in a T2D-induced mouse model, fenofibrate (a PPARα agonist) was able to inhibit NLRP3 inflammasome and the subsequent IL1-β production through a ROS-dependent mechanism that implies thioredoxin-interacting protein (TXNIP), improving the angiogenic epithelial precursor cells ability [175]. Altogether, these results indicate that the NLRP3 inflammasome can be regulated by distinct molecules under different metabolic states, and that these effects are mainly achieved through IL-1β production.

The role of IL-18 (also produced upon inflammasome activation) in obesity, remains controversial [176]. In this sense, serum levels of IL-18 correlate with insulin resistance and metabolic syndrome components. Strikingly, IL-18 has marked anti-obesity effects as interleukin administration prevents weight gain, while IL-18 loss is associated with increased adiposity and insulin resistance in mice models [177, 178]. Murphy and cols. demonstrated that IL-18 production relies on NLRP1 inflammasome. Using mice models, they demonstrated that loss of *Nlrp1* decreased IL-18 levels, lipolysis, and led to obesity and metabolic syndrome [179]. Noteworthy, Raut and coworkers demonstrated that leptin, one of the major adipokines produced in obese conditions and related to tumor growth, is able to activate NLRP3 inflammasomes in MCF-7 breast cancer cells [180]. This induction, which courses with IL-1β production, and overexpression of NLRP3 and ASC component, was ROS dependent (in a mechanism involving NADPH oxidase) and was further corroborated in xenograft models. Raut and coworkers also demonstrated that adiponectin, an adipokine severely diminished in obesity that exerts anti-inflammatory and antitumor actions, completely suppressed leptin-induced NLRP3 inflammasome activation [181]. Interestingly, leptin has been shown to activate also NLRP3 inflammasome in macrophages increasing IL-18 secretion [182]. Altogether, these results unveil the complex relationship between adipokines and inflammasomes, which could be responsible, at least in part, for the inflammatory responses triggered in the AT under obesity conditions.

Investigation about other inflammasomes types in the obesity field is limited. *Aim2* deficient mice exhibit impaired glucose homeostasis, upregulation of pro-inflammatory pathways, augmented adipogenesis, and inflammation in the AT with an increased rate of monocyte infiltration, highlighting the role of AIM2 inflammasomes in energy metabolism homeostasis [183]. In a novel study, NLRC4 inflammasome has been associated to breast cancer progression in obesity conditions. Specifically, *NLRC4* mRNA is elevated in normal breast tissues from patients with obesity and positively correlates with BMI. Moreover, it has also been reported that obesity-induced NLRC4 inflammasome in tumor-infiltrating myeloid cells promotes angiogenesis in diet-induced obese mice through IL-1β, and that such activation would finally lead to increased *Vegfa* expression in surrounding adipocytes [184]. In fact, angiogenesis has also been suggested to be disturbed through NLRP3-related mechanisms in cardiovascular diseases influenced by short fatty chain acids [185]. In line with these results, it has been shown that, in an obese state, breast tissue-associated adipocytes recruit and activate macrophages through an IL-1β dependent mechanism [186]. Furthermore, a protective role has been suggested for NLRP12 inflammasome whose expression is reduced in the AT of humans with increased BMI. Also, *Nlrp12* deficient mice showed increased weight gain, macrophages infiltration, and adipose tissue inflammation [187].

Furthermore, in this specific context, certain coenzymes have demonstrate to exert advantageous effects. Specifically, coenzyme Q10, which is considered to be a potential anti-cancer agent, shown to be beneficial when administered daily to overweight and obese patients with diabetes, improving glucose and lipid homeostasis [188]. However, weak associations have been found in PCa plasma patients [189]. Another coenzyme, Q0 was able to decrease HIF-1α expression, to ameliorate NLRP3-mediated inflammation, and suppress the EMT/metastasis and the metabolic rewiring in breast cancer [190].

Along with increased systemic inflammation levels, specific organs have also been related to inflammatory responses under obesogenic conditions. For example, inflammation in the liver and pancreatic islets is also evident in individuals with obesity [165]. These ‘from systemic to localized’ inflammatory responses could also affect the prostate under obesogenic conditions, leading, in the last instance, to prostate alterations, and malignancies. In this context, the relationship between obesity and some typical hallmarks of cancer, such as angiogenesis, EMT, genomic instability, and inflammation has been already described [191]. However, the casual link between obesity and PCa is more controversial. In fact, unlike other tumor pathologies, PCa is energetically more dependent on lipid β-oxidation than on glucose oxidation, thus exhibiting a unique profile far from the "Warburg" glycolytic phenotype compared with that found in the majority of the tumors [192]. Concordantly, PCa cells show a high rate of *de novo* synthesis of fatty acids (partially driven by AR activation), and active lipid uptake, which make even lipid accumulation visible intracellularly [193]. In fact, various key lipidic enzymes (i.e., ACLY, ACC, and FASN) are markedly overexpressed in PCa [194]. Although this peculiarity could be understood as a point to exploit, a recent review article concludes that lipid-targeted drugs are unlikely to replace current highly effective treatments for metastatic PCa but can be used in combination to improve response rates and longevity of cancer control [195].

Despite this, the causal relationship between obesity—whose origin lies in adipose tissue which could be a direct source of metabolic fuel—and PCa is still a matter of debate. A study affirms that the higher BMI, the lower risk of PCa diagnosis, but the risk of PCa mortality increases in such cases [196]. In consonance, another work suggest that the median diagnostic age decreases with increasing BMI, and that overweight and obese patients are more likely to have and advanced or metastatic PCa at diagnosis [197]. Parallelly, they also demonstrated that PSA levels tend to become lower in the higher BMI groups, a possible explanation for the late diagnosis [197]. In this regard, it has also been proved that a further increase in the BMI (above 28) is inversely associated with the likelihood of surgical treatment compared with radiotherapy, although no evidence of worse functional outcomes was found in obese patients undergoing definitive treatment nor remained on active surveillance [198]. However, through genetic profiling-based approaches, it has been also concluded that adiposity is unlikely to influence PCa via metabolic factors [199]. *In vitro*, a recent study demonstrated that conditioned media derived from the 3T3-L1 adipocyte line was able to elicit profound phenotypic changes in DU145 and PC-3 models, i.e., elevated stemness markers, increased EMT, clonogenic, prostatosphere formation, and invasiveness capacity, as well as showing evidence of chemoresistance to docetaxel and cabazitaxel [200].

In this scenario, during the last years, a specific fat tissue depot, the periprostatic adipose tissue (PPAT) has attracted attention in this research field. PPAT directly surrounds the prostate gland and could act as an energy reservoir for growing tumors, as well as a local source of factors [201]. Remarkably, a significant positive correlation has been found between BMI and PPAT thickness [202]. Thus, this tissue could represent a crucial player in the connection between systemic low-chronic inflammation associated with obesity and localized inflammation of the prostate, which could contribute to PCa development [203]. A detailed transcriptomic PPAT profiling study suggests a hypoxic state that courses with HIF2-α overexpression [204]. Concordantly, various inflammatory-related pathways are also overrepresented, finally leading to a drastically altered PPAT extracellular matrix remodeling, deposition, and composition [204].

**Fig. 3:**
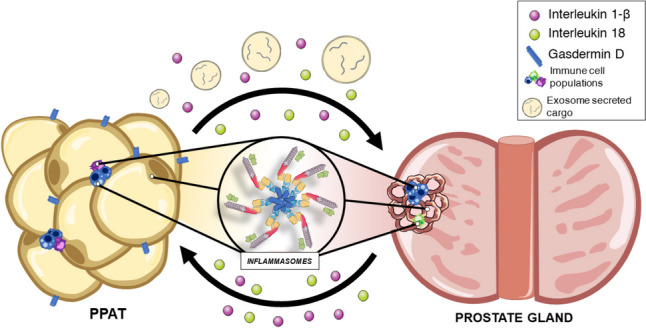
*Interaction between periprostatic adipose tissue, prostate gland, and inflammasomes*. Different types of functional inflammasomes have been detected in multiple cell types in periprostatic adipose tissue (PPAT) and the prostate gland. Crosstalk between both tissues could be mediated reciprocally by surrounding proinflammatory molecules produced by inflammasomes, and miRNAs included in adipose tissue derived exosomes

Another example is concerning IL-6, which is associated with the most aggressive phenotypes of PCa and may be involved in metastatic process. In addition, IL-6 is involved in CRPC development through accessory activation of AR [140]. It is also known that M1 macrophages aggregate around necrotic adipocytes in inflamed tissue, forming ‘crown-like structures’ and producing IL-6 [205]. High IL-6 levels have been found in PPAT and may be involved in a malignant environment that favors the development of more aggressive PCa [206]. Therefore, for all the information stated above, it is possible that cytokine signaling between the PPAT, and the prostate may be controlled, at least in part, by inflammasomes activation (Fig. 3).

### The influence of miRNAs in adipose tissue, prostate, and inflammatory response

In the context of AT and prostate, and how the metabolic status could influence the potential tumor malignancy through inflammasome-related mechanisms, the exchange of information via exosomes and their cargo, may be also of great importance (Fig. 3). In fact, it has been shown that the number and the specific content of exosomes released from AT significantly change in patients with obesity [207]. Specifically, it has been demonstrated that different miRNAs may play a key role in this scenario. For example, leptin levels have been shown to influence PCa through the downregulation of miRNA-628, which is able to inhibit proliferative, migratory, invasive, and spheroid-forming capacity *in vitro* [208]. A comprehensive revision that compiled the miRNAs controlled by three of the main adipokines (i.e., adiponectin, leptin, and resistin) that influence certain tumor diseases has been published [209]. Notably, it has been reported that miR-7a-2 and miR-1977 are two of the critical transcriptomic factors (many of them related to the immune and inflammatory response) that contribute to a specific and discriminating PPAT expression signature between obese and overweight patients with PCa [201].

Simultaneously, those secreted factors (adipokines and/or miRNAs) released by AT, whose levels are influenced by metabolic status, could also affect the endogenous levels of miRNAs produced and released by the prostate, constituting an additional complexity layer between AT, obesity, and PCa [209, 210]. In this context, the uptake of tumor-secreted exosomes by stromal cells induces a reprogramming that contributes to the formation of new tumor cells [211]. Some examples include: miR-205, which has been reported to be regulated by the activation of the NLRP1-inflammasome locus snpRNAs and the subsequent control of 8q24-locus expression, establishing a complex epigenetic regulatory network, which ultimately modulate the expression of a footprint of genes that includes the tumor suppressor *PTEN* [212]; miR-17/92, whose levels are modulated by testosterone and 1,25-dihydroxyvitamin D_3_, and affect PPARα transcript stability promoting lipid synthesis and energy storage in PCa cells [213]; miR-21, which is significantly associated with PCa recurrence after radical prostatectomy in BMI-dependent manner [214]; or miR-139-5p, which is directly regulated by the fat mass and obesity-associated (FTO) protein, and exerts tumors modulating functions through ZNF217 [215].

Besides acting as potential messengers, miRNAs have also become a potent diagnostic and prognostic tool in PCa, becoming a highly exploited source of biomarkers in recent years. For example, it has been proved that plasma levels of let-7c, let-7e, let-7i, miR-26a-5p, miR-26b-5p, and, especially, miR-18b-5p and miR-25-3p showed a clear discriminatory capacity between PCa with elevated PSA levels among patients with BPH [216]. In urine, the combined measurement of miR-21, miR-19a, and miR19b also showed a higher prediction value that overcomes the PSA-based detection method [216]. Recently, the analysis of the whole plasma miRNome from PCa patients pointed out miR-107 as the best discriminatory miRNA between PCa and control patients, showing even better discrimination capacity when only patients with obesity were considered [33]; likewise, miR-4454 levels are highly altered and associated with metabolic disorders such as obesity and insulin metabolism in PCa [217].

Considering that functional inflammasomes have been found in visceral fat tissue and prostate epithelial cells, and that a wide variety of immune infiltrate cells are present in both types of tissues, inflammasome activation could represent a chicken-and-egg situation, enabling to establish a bidirectional crosstalk and self-amplifying signaling driving PCa development in patients with obesity.

## Future perspectives

The compiled evidences suggest that inflammasome may be considered a key target in inflammation-related diseases. In fact, several initiatives focused on the blockade of inflammasome have been recently implemented in diverse areas. Thus, although the possibilities are limited and more selective drugs are still under development, various treatments have been already identified to target inflammasomes components [218].

Among them, the compound termed MCC950, a daily sulfonylurea-containing molecule, is considered the most potent and selective NLRP3 inflammasome inhibitor [219]. MCC950 treatment improved hematopoiesis in a myeloid leukemia mouse model and human-derived samples from myelodysplastic syndrome patients [220, 221], and to reduce tumor burden in squamous cell head and neck carcinoma mouse model [222], though research in this field is still limited.

A special mention of caspase-1 inhibitors should be made. Molecules such as VX-765 (an oral pro-drug) have been demonstrated to notably diminish the release of IL-1β and IL-18, yet its clinical use is still under investigation [223]. Another group of strategies comprises those based on exploring the modulation of the up and down-stream pathways of the inflammasome, by targeting priming and activating signals or by directly blocking the downstream IL-1β pathway. We have summarized in Table 2 the current information regarding inhibitors that target structural NLRP3 inflammasome components.

**Table 2:** List of NLRP3 Inflammasome Inhibitors

**Component**	**Target**	**Effect**	**Ref**
**Parthenolide**	NLRP3	Blocks ATPase activity	[225]
**Bay 11–7082**	NLRP3	Blocks ATPase activity	[225]
**CY-09**	NLRP3	Blocks ATPase activity	[226]
**Dapansutrile**	NLRP3	Blocks ATPase activity	[227]
**INF148/INF120/INF156/INF172**	NLRP3	Blocks ATPase activity	[228]
**MNS**	NLRP3	Blocks ATPase activity	[229]
**Oridonin**	NLRP3	Prevents oligomerization	[230]
**Tranilast**	NLRP3	Prevents oligomerization	[231]
**11Cha1**	ASC	Prevents oligomerization	[232]
**MCC950**	ASC	Blocks ATPase activity	[233]
**Ac-YVAD-CHO**	CASP1	Blocks protease activity	[234]
**Belcanasan**	CASP1	Blocks protease activity	[223]
**Parthenolide**	CASP1	Blocks protease activity	[225]
**Pralnacasan**	CASP1	Blocks protease activity	[235]
**Thalidomide**	CASP1	Blocks protease activity	[236]

Remarkably, some clinical trials have been performed concerning inflammasomes and obesity. The NCT05071391 trial aimed to determine the relationship between autophagy and inflammasomes regulation in the pathogenesis of obesity and related comorbidities; however, no published results are available yet. On the other hand, the NCT04315376 trial evaluated the effect of a hypocaloric diet on inflammasome adaptor ASC (22), alone or in combination with physical exercise-based intervention (15) in two different groups of patients with obesity showing its potential as a follow-up biomarker in such types of interventions [224]. Additionally, three other inflammasome-related clinical trials are in the recruiting phase. The clinical trial NCT04814147 intends to study the inflammasomes after sleeve gastrectomy intervention in women with obesity; the trial NCT04275674 aims to study the macrophages inflammasome in a cohort composed of 200 women with obesity and the likely connection with miscarriages; and finally, the global aim of NCT04485871 is to explore the pivotal role of NLRP3 inflammasome in T2D-associated inflammation through an ω-3 dietetical supplementation.

However, to the best of our knowledge, no studies have been made to explore the modulation of inflammasomes activities in the context of cancer and especially PCa. Although the therapeutic possibilities of inflammasome cascade inhibition are promising, the available results should be taken carefully, as blocking central inflammatory mediators could be a double-edged sword. In this regard, a thoroughly investigation about possible risks/benefits should be accomplished before this type of therapies could be used in the future.

## Conclusions

Inflammasomes are macromolecular structures able to regulate the inflammatory response through the production of pro-inflammatory molecules. Although inflammasomes were initially associated with immune cell populations, recent studies have demonstrated that functional inflammasomes could be assembled under certain conditions (e.g., hypoxia, lesions, or infections) in human prostate cells. However, in the case of prostate cancer, the available information is limited and only a few types of inflammasomes have been explored. On the other hand, evidence connecting the role of inflammasomes and weight-related disorders, such as obesity, is more solid due to the marked inflammatory nature of these conditions, being also active in adipocytes and in the immune cells infiltrating the adipose tissue. In both pathological states, current information points that IL-1β and NF-kB are central players orchestrating an inflammatory response, although the investigation in this regard is in the initial steps and much more effort is needed to unveil the connection between prostate cancer, obesity, and inflammation. Therefore, understanding the complex role that inflammasomes could be playing, and considering the availability of drugs that can specifically modulate them, may represent a novel and valuable source of therapeutic tools and strategies of action.

## Abbreviations

AT: Adipose Tissue
ATMs: Adipose Tissue Macrophages
AR: Androgen Receptor
BPH: Benign Prostate Hyperplasia
BMI: Body Mass Index
CARD: Caspase Activation and Recruitment Domain
CRPC: Castration Resistant Prostate Cancer
CLRs: C-type lectin receptors
DAMPs: Damage-Associated Molecular Patterns
EMT: Epithelial to Mesenchymal Transition
HIF-1α: Hypoxia-inducible factor 1-alpha
IL-18: Interleukin-18
IL18BP: Interleukin-18 Binding Protein
IL-1β: Interleukin-1β
NLRs: Nucleotide-binding Oligomerization Domain-like receptors
PYD: PAAD/DAPIN Domain
PAMPs: Pathogen-Associated Molecular Patterns
PRRs: Pattern Recognition Receptors
PPAT: Periprostatic Adipose Tissue
PIA: Proliferative Inflammatory Atrophy
PCa: Prostate Cancer
PIN: Prostatic Intraepithelial Neoplasia
ROS: Reactive Oxygen Species
RLRs: Retinoic acid-Inducible Gene 1
SNPs: Single Nucleotide Polymorphisms
SVF: Stromal-Vascular Fraction
TLRs: Toll-like receptors
T2D: Type 2 Diabetes
